# Characterization of the Optical Properties of Photoluminescent Turbid Media Using an Integrating Sphere and Monte Carlo Simulations

**DOI:** 10.3390/ma17246072

**Published:** 2024-12-12

**Authors:** Joachim Jelken, Thomas Brall, Philip Gelbing, Florian Foschum, Alwin Kienle

**Affiliations:** Institut für Lasertechnologien in der Medizin und Meßtechnik, Universität Ulm, Helmholtzstr. 12, 89081 Ulm, Germany

**Keywords:** integrating sphere, turbid media, optical properties, photoluminescence, Monte Carlo simulation

## Abstract

In this paper, we report on the measurement of the optical properties (absorption and scattering coefficients) of photoluminescent turbid media using a homemade integrating sphere setup equipped with a tunable monochromatic light source. The hemispherical reflectance and transmission data are analyzed with the radiative transfer equation using a Monte Carlo simulation-based lookup table to obtain the optical properties of the sample. The results are compared with the optical properties received from a classical integrating sphere setup equipped with a broadband white light source. The additional light of the photoluminescence generates artifacts within the optical properties, which are not present using a monochromatic light source. Additionally, a batch of samples with a broad range of scattering coefficients and dye concentrations were prepared and characterized with the aforementioned setup. The findings can help to generate a digital twin with the optical properties of the sample, which improves the physically based rendering and the design of, e.g., white-light LEDs. Dental restoration and photodynamic therapy also benefit from determination of the optical properties of photoluminescent turbid media.

## 1. Introduction

Knowledge of the optical properties of turbid media, e.g., scattering and absorption coefficients, has gained more and more attention in recent years because of its manifold applications. The data are used in medicine, e.g., for cancer diagnostics [1,2,3,4], or in photodynamic therapy [5,6], to optimize sensors for autonomous driving by simulating fog [7,8] or for the photorealistic physical rendering of objects [9,10,11]. Another branch is the fabrication of optical phantoms with the optical and mechanical properties as its real existing counterpart [12,13,14]. These phantoms are used to train instruments, e.g., for the disease diagnostics of plants or humans. The absorption and scattering coefficients are also important for the visual impression of dental restoration material [15,16,17]. Therefore, the precise determination of the optical data is crucial. For transparent samples, the absorption coefficient is obtained from the Lambert–Beer law, while for turbid media the measurement is more challenging. Here, light scattering effects have to be considered. Usually, for such kinds of measurements an integrating sphere equipped with a white light source, e.g., a halogen lamp, and a spectrometer are used. After the measurement, the optical properties are obtained from an inverted Monte Carlo simulation [18,19,20]. However, this limits the variety of quantifiable samples to non-luminescent samples, because the detector cannot distinguish between elastically scattered light and the photoluminescence of the sample. The additional light of the photoluminescence results in artifacts within the measured optical properties if not considered for in the evaluation. This problem was overcome by adding a tunable monochromatic light source to the setup. In these approaches, the sample was located within the sphere [21,22,23,24] or a double sphere configuration [25,26,27,28] was used. This made the sample handling more complicated and the Monte Carlo simulation more complex because additional sample holders and baffles had to be considered.

In our approach, we use a single integrating sphere with a tunable monochromatic light source and a spectrometer for the detection, and the sample is located outside the sphere. This permits the determination of the total hemispherical reflectance, the total hemispherical transmittance and the photoluminescence in transmission and the reflection geometry for samples with a broad range of optical properties. The comparison of the recorded reflectance and transmission with a lookup table based on an inverted Monte Carlo simulation provides the optical properties of the photoluminescent sample. Thus, the setup can help to optimize, e.g., the design of white-light LEDs, by creating a digital Monte Carlo twin that models the scattered light transport through the active area of the LED based on the determined spectral-resolved optical scattering and absorption coefficients. In general, white-light LEDs are composed of several different types of phosphor, which act as light scatterers, and their composition is crucial for the emission spectrum and quantum efficiency [29,30].

## 2. Materials and Methods

### 2.1. Experimental Setup

**Figure 1:**
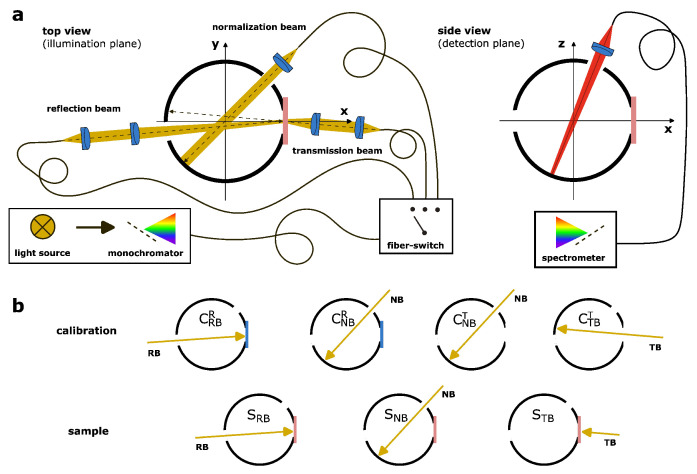
Sketch of the experimental setup (**a**) and of the measurement routine (**b**). A mirror (in blue color) was added at the sample port for the calibration measurement and was later replaced by the sample (in pink color).

The optical properties, such as the effective scattering coefficient ($\mu_{s}^{\prime }$) and absorption coefficient ($\mu_{a}$), of photoluminescent samples were measured for the spectral range between 320 nm and 1000 nm using a homemade integrating sphere setup, based on a 3D-printed sphere, a tunable monochromatic light source and a CCD spectrometer. The fabrication of the sphere is described elsewhere [31]. In short, a sphere with an inner diameter of 150 mm and a wall thickness of 3 mm was printed with a commercial 3D printer (Ultimaker 3.0, Ultimaker B.V., Geldermalsen, The Netherlands) using black polylactic acid filament and a filling factor of 0.7. The design was kept clean, i.e., no additional baffles were added, to optimize the Monte Carlo simulation of the light propagation within the sphere. Three illumination ports and one detection port were added to the layout of the sphere, see Figure 1a. The port for the transmission beam (sample port, 25 mm in diameter) and the port of the reflection beam (20 mm in diameter) are in the equatorial plane of the sphere and ensure an illumination of the sample of 8° relative to the surface normal. The last illumination port (20 mm in diameter) is used for the normalization beam, which illuminates only the inner wall of the sphere, see Figure 1a. The detection port (20 mm in diameter) is located near the north pole of the sphere and collects the light from an area (6 mm in diameter) close to the south pole of the sphere. Directly after printing, the inner part of the sphere was leveled with epoxy resin and ground with fine sandpaper. Afterwards, a white acrylate universal primer was applied until a total hemispherical reflectance of 80–85% was achieved. In a second step, a barium sulfate coating by Gigahertz-Optik (Türkenfeld, Germany) was applied until saturation in the wall reflectance was reached. The sample was mounted to the sample port with leaf springs. The light of a laser-pumped plasma light source (Isteq XWS 65, spectral range 190–2500 nm) was wavelength-selected by a monochromator ((Mountain Photonics, Landsberg am Lech, Germany), Hyperchromator II, spectral range 250–1600 nm, two gratings 300 lines/mm and 1250 lines/mm) and coupled into an optical fiber ((Gulf Photonics, Oldsmar, FL, USA) multimode, core diameter 600 micron, 0.22 NA, spectral range 200–2100 nm). The other end of the fiber is connected to a homemade three-port fiber switch. The rotation of an internal mirror with a stepper motor selects the exit port. To each of these three ports, a fiber is connected, with the same parameters as described before, used as transmission beam, reflection beam and normalization beam. At the end of the fibers, apertures and lenses are used to project the fiber ends onto the wall of the integrating sphere, the surface of the sample or calibration standard. The beam diameter is set to 5 mm at the measurement spot. A $CaF_{2}$ lens (*f* = 25 mm) is connected to the detection port and projects the top hat intensity profile of the sphere wall onto the end of an optical fiber (Thorlabs, Newton, NJ, USA) multimode, low OH, core diameter 1000 micron, 0.39 NA, spectral range 400–2200 nm). The other end of the fiber is connected to a CCD spectrometer (Ocean Optics, Orlando, FL, USA) Ocean HR 6 XR, 300 lines/mm grating, 100 micron entrance slit, spectral range 200–1100 nm, resolution 4 nm). The whole setup is mounted on an optical table and encapsulated from ambient light by a black hardboard enclosure.

### 2.2. Sample Preparation

The samples were prepared by mold casting of a silicone rubber (Wacker Chemie, Munich, Germany, Elastosil M 4641 A/B). Rhodamine 6G (Lambda Physik AG, Goettingen, Germany, Lambdachrome Laser dye LC 5900) was first dissolved in ethanol at a concentration of 0.1 mg/mL and afterwards added to the base elastomer (4 wt%). The two components were mixed in two steps: First by manual stirring followed by adding 8 g of grinding beads (SIGMUND LINDNER GmbH, Warmensteinach, Germany, Silibeads ZY 6.0 2.8–3.2 mm) and using a centrifuge vortex mixer (Hauschild GmbH, Hamm, Germany, SpeedMixer DAC 800.2 VAC-P) for 4 min at 1000 rpm. In the last step, vacuum was applied to remove any residual ethanol. In another process, ZrO_2_ particles of 800 nm in diameter (US Research Nanomaterials Inc., Houston, TX, USA) were mixed into the base elastomer, following the same protocol. Additionally, a dispersing additive (DISPERBYK-2155, BYK Chemie, Wesel, Germany) with a concentration of 1.3 wt% was put in, as well. The premixed R6G emulsion and ZrO_2_ dispersion were added to the base elastomer in a desired ratio to adjust the R6G and particle concentration. The mixture was then dispersed using a speedmixer. Finally, a curing agent (component B) was added in a ratio of 1:10 to the base elastomer followed by 4 min mixing in vacuum to remove trapped air and solvent bubbles. The emulsion was poured into molds of different heights to adjust the sample thickness and then cured for 24 h at room temperature.

### 2.3. Measurement Routine

The measurement procedure was split into two parts, the calibration and the sample measurement. At the start of any measurement, the light source and the spectrometer were turned on and a waiting period of 30 min was applied to let the temperature of the light source and the spectrometer stabilize. For the calibration measurement, a reflectance standard, i.e., a mirror with a known spectral reflectance, was mounted to the sample port and the fiber switch was moved into the reflection beam ($C_{RB}^{R}$) position, meaning that the mirror was directly illuminated (under an angle of 8°) through the reflectance port (see Figure 1b). Afterwards, the wavelength of the illumination was scanned from 320 nm to 1000 nm in steps of 10 nm and a complete spectrum was recorded for each excitation wavelength. The spectral acquisition time was adjusted for each spectral range, as a result of different intensities provided by the light source, the spectral diffraction efficiency of the grating within the monochromator and the spectral sensitivity of the spectrometer. In the spectral range from 320 to 340 nm, the acquisition time was set to 100 ms, from 350 to 600 nm to 50 ms, from 610 to 690 nm to 100 ms and from 700 nm to 1000 nm to 200 ms. The change in the acquisition time results in a better signal-to-noise ratio, which reduces the relative error in the total hemispherical reflectance and transmittance. The full width at half maximum (FWHM) of the excitation beam was set to ≈ 7 nm by adjusting the slit width of the monochromator. An average of ten spectra were taken to improve the signal-to-noise ratio. Prior to the measurement, dark spectra were obtained and subtracted from the recorded data. Afterwards, the fiber switch was moved into the normalization beam ($C_{NB}^{R}$) position and the measurements were repeated. The normalization beam corrects minor deviations from the ideal Lambertian angular distribution of the sphere wall. After these two initial measurements, the mirror at the sample port was removed and the measurements with the normalization beam were repeated, but this time with an open sample port ($C_{NB}^{T}$). The final measurement of the calibration procedure was the transmittance measurement. Therefore, the fiber switch rotated into the third position so that the sphere was illuminated through the sample port ($C_{TB}^{T}$). After the calibration, the sample was mounted to the sample port and the measurement routine was repeated, indicated as $S_{RB}$, $S_{NB}$ and $S_{TB}$. Note that just one normalization beam illumination was needed for the sample measurement. The whole setup, including the monochromator, fiber switch and spectrometer, was controlled by an automated C++ software (Visual C++ 2022, Microsoft, Redmond, WA, USA) routine. The total hemispherical reflectance R and transmittance T were calculated from the recorded data by integration over the excitation peak (elastic light scattering, 10 nm spectral range) in the spectra and using the following equations [18,31]:

(1) $$R=\frac{S_{RB}C_{NB}^{R}}{S_{NB}C_{RB}^{R}}\rho_{cal};\;T=\frac{S_{TB}C_{NB}^{T}}{S_{NB}C_{TB}^{T}}.$$

Here, $\rho_{cal}$ is the hemispherical reflectance coefficient of the mirror at a specific wavelength and was determined by recording the intensity reflected from the surface relative to the incident intensity at an angle of 8° as a function of wavelength. Hence, the optical properties of the sample were determined by a two-dimensional interpolation algorithm comparing the R and T of an analytically corrected and inverted lookup table (LUT) with the measurement. The LUT was calculated with the radiative transfer equation using a Monte Carlo method, which considers the effective reflectance of the sphere walls, port losses and the part of the signal that directly illuminates the detector, resulting in an effective reflected and transmitted signal [18,31]. Therefore, the refractive index (*n*), asymmetry factor (*g*), sample thickness (*d*) and a suitable subdivision in the optical parameters $\mu_{s}^{\prime }$ and $\mu_{a}$ have to be selected accordingly. For the evaluation of the integrating sphere measurements, an average asymmetry factor of *g* = 0.75 was used and the refractive index of the silicon rubber matrix was measured with an ellipsometer equipped with a white light source and a spectrometer. The asymmetry factor *g* was calculated from Mie theory by considering the spherical shape of the ZrO_2_ particles and their size distribution as well as the refractive index of the silicone matrix and of the particles. It is a common measure for how much forward direction is retained after a single scattering event. An asymmetry factor of *g* = 0.75 is associated with mainly forward scattering. Therefore, our setup determines the optical properties of photoluminescent turbid media and records a complete photoluminescence spectrum of the sample (in the transmission and reflectance directions) for each excitation wavelength at the same time.

## 3. Results

**Figure 2:**
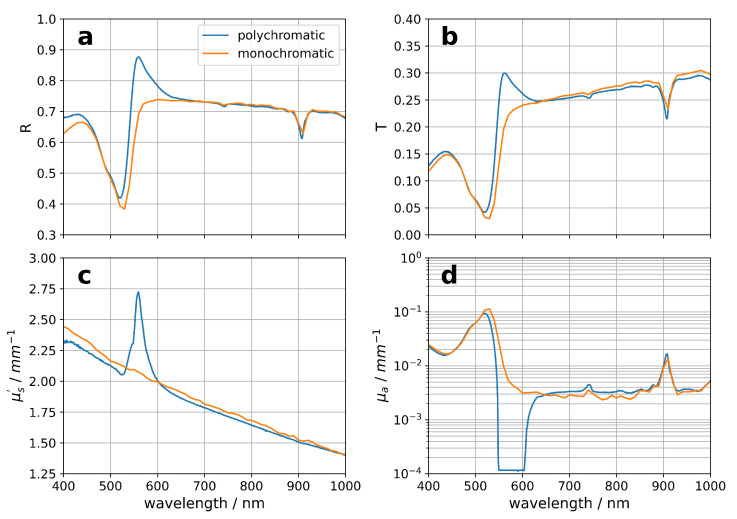
Comparison of the integrating sphere measurements using a mono- and polychromatic light source. The recorded total hemispherical reflectance R is shown in (**a**) and the transmittance T in (**b**). The scattering coefficient $\mu_{s}^{\prime }$ (**c**) and the absorption coefficient $\mu_{a}$ (**d**) show artifacts for polychromatic illumination of the sample.

In a classical integrating sphere approach, polychromatic illumination of the sample, provided by, e.g., a halogen light source, is applied and the scattered light of the transmission, reflectance and normalization beams is detected with, e.g., a CCD spectrometer. In the case of a photoluminescent sample, the excitation beam will cause photoluminescence, which results in an additional signal. Therefore, the calculated values of the reflectance R and transmittance T are mistaken, because the analysis cannot distinguish between the elastic scattered light and the photoluminescence and adds up the two signals. As a consequence, the corresponding optical properties, determined from the false R and T values, are wrong. This problem is overcome in our approach due to the recording of a spectrum for each excitation wavelength. Here, the scattered light is separated from the photoluminescence signal and the integral over the elastically scattered light is taken for the calculation of the R and T values. Figure 2 compares the optical properties obtained with a classical integrating sphere setup based on a polychromatic illumination and the results using an integrating sphere with monochromatic illumination. Therefore, a Rhodamine 6G (R6G) sample with ZrO_2_ particles as scatterers in a polymer matrix was prepared. R6G was chosen because it is well studied in the literature, showing a strong absorption in the visible range and a photoluminescence with a maximum at 548 nm with a high quantum yield. The absorption maximum of R6G at 520 nm is clearly visible in the recorded R and T values (see Figure 2a,b). The comparison of the two data sets shows an additional peak at 560 nm for the R and T of the classical integrating sphere setup based on polychromatic sample illumination. The reason for this extra peak is the photoluminescence of R6G, which is not distinguished from the elastically scattered light. Therefore, the corresponding scattering coefficient $\mu_{s}^{\prime }$ (Figure 2c) and absorption coefficient $\mu_{a}$ (Figure 2d) also show artifacts, because the wrong hemispherical reflectance and transmittance are used for the comparison with the lookup table. Thus, this configuration is not suitable for the precise determination of the optical properties of photoluminescent turbid media. The appropriate method for such kinds of samples is the stepwise monochromatic illumination and the recording of a complete scattering and emission spectrum at each excitation wavelength.

**Figure 3:**
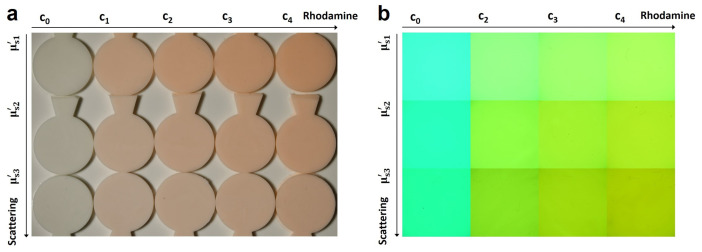
Optical images of the samples with different optical properties. In (**a**), a photograph of the samples under white-light illumination from the top is shown. The R6G concentration is increasing from left to right and the scattering coefficient from top to bottom. Photographs of the samples under monochromatic illumination (λ = 528 nm) from below are shown in (**b**). An increase in photoluminescence is observed with an increase in the R6G concentration, which changes the color from turquoise to green.

To test the new developed setup for its performance, a 5 × 3 matrix of samples was prepared, with four different R6G concentrations ($c_{1}$ = 2.66 wt%, $c_{2}$ = 4 wt%, $c_{3}$ = 5.33 wt% and $c_{4}$ = 6.66 wt %), one sample without R6G ($c_{0}$) and three different concentrations of ZrO_2_ particles. Additionally, the sample thickness was varied between 1 mm, 2 mm and 4 mm in order to tune the optical thickness of the sample, which extends the range of measurable optical properties. An optical image of the samples under white-light illumination from the top is shown in Figure 3a (sample thickness 4 mm). Here, the R6G concentration is increasing from left to right and the scattering coefficient is increasing from top to bottom. In Figure 3b, photographs of the samples under monochromatic illumination from the back with a green LED (λ = 528 nm) are shown. The sample thickness was reduced to 2 mm for these measurements for a better light transmission. The color transitions from turquoise to green with an increase in the R6G concentration.

**Figure 4:**
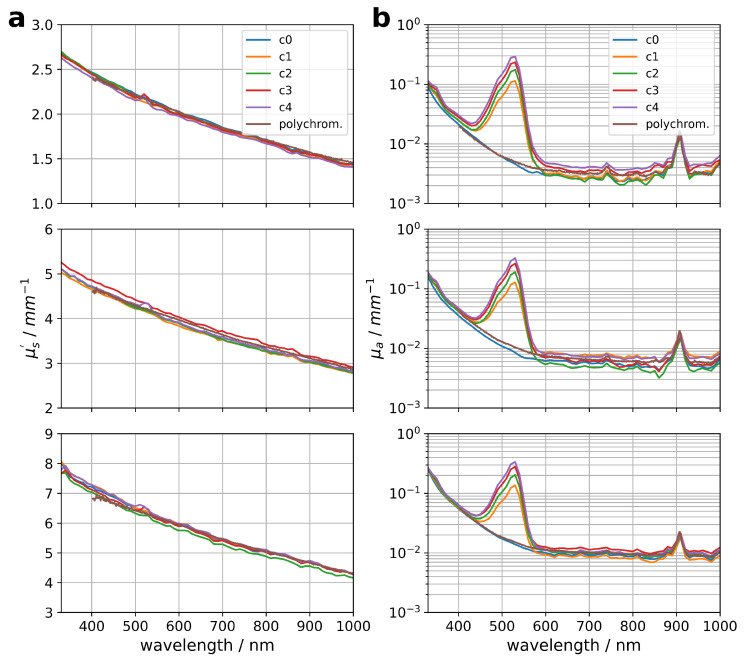
Optical properties of samples with different R6G and ZrO_2_ particle concentrations. The scattering coefficient $\mu_{s}^{\prime }$ is shown on the left (**a**) and the corresponding absorption coefficient $\mu_{a}$ on the right (**b**). The optical properties measured with polychromatic light and on a sample without R6G ($c_{0}$) are also presented for comparison.

The optical properties of these samples are shown in Figure 4. The scattering coefficient (Figure 4a) of the samples range from $\mu_{s}^{\prime }$ = 2 mm^−1^, 4 mm^−1^ to 6 mm^−1^ at 600 nm for the different particle concentrations and, as expected, only a small variation for the corresponding samples with a different R6G concentration is observed. The sample without R6G was also characterized with a classical integrating sphere setup for the purpose of comparison and validation. The results are in agreement in terms of shape and absolute value with the results for a monochromatic illumination of the sample. In Figure 4b, the corresponding absorption coefficient $\mu_{a}$ is shown. It is observed that the absorption coefficient varies over several orders of magnitude, from 2 $\times \;10^{-3}$ mm^−1^ to 2 $\times \;10^{-1}$ mm^−1^. The R6G absorption peak at 525 nm scales with the R6G concentration. The comparison of the absorption coefficient of the sample without R6G with the data recorded with a classical integrating sphere setup shows a match in shape and absolute value. It should be noted that making the sample thickness too thin causes the optical path length to be too short to allow precise measurements of the absorption. In contrast, preparing a sample with a high R6G concentration so that the optical attenuation is too large will result in insufficient light reaching the detector.

**Figure 5:**
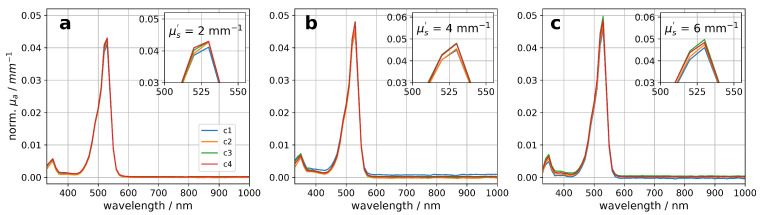
R6G concentration normalized absorption coefficient for different concentrations of R6G and particles. In (**a**), the results for the scattering coefficient of $\mu_{s}^{\prime }$ = 2 mm^−1^, in (**b**) for $\mu_{s}^{\prime }$ = 4 mm^−1^ and in (**c**) for $\mu_{s}^{\prime }$ = 6 mm^−1^ are shown. The absorption coefficient of the sample without R6G ($c_{0}$) was subtracted from the data prior normalization.

The spectral-resolved absorption coefficient of R6G was calculated by subtracting the absorption coefficient of the sample without R6G ($c_{0}$) from the recorded data. Figure 5 shows the absorption coefficient normalized by the R6G concentration within the sample for four R6G concentrations and three particle concentrations. It is observed that the absorption coefficient scales linearly with the R6G concentration for all three studied particle concentrations (see Figure 5a–c).

## 4. Conclusions

In this paper, we measured the optical properties in the UV–VIS–NIR range of photoluminescent turbid samples with a broad spectrum in their scattering and absorption coefficients. A new experimental setup, based on an integrating sphere and a tunable monochromatic light source, was developed. In contrast to other approaches, the sample is mounted outside the sphere, so that no sample holder or additional baffles inside the sphere are needed. The recorded hemispherical transmission and reflectance data were compared with an inverted LUT generated by a Monte Carlo simulation of the radiative transfer equation to obtain the optical properties of the samples. The results were correlated with those obtained by the classical approach of an integrating sphere with polychromatic illumination. It was found that the additional light of the photoluminescence during polychromatic illumination results in artifacts within the recorded hemispherical reflectance and transmittance. The wrong R and T values in the spectral range of the photoluminescence are assigned by the lookup table to false optical properties. These artifacts vanish using monochromatic sample illumination, because of the selective excitation and the evaluation of just the elastic scattered light with the help of a spectrometer. These findings can help in the determination of the quantum efficiency of R6G in turbid media using this measurement geometry, which is usually challenging. The design and optimization of white-light LEDs also benefits from this work. Here, based on a Monte Carlo simulation and the recorded optical data, a digital twin of the sample can be generated, which helps to optimize the efficiency of a white-light LED. The determination of the optical properties of photoluminescent turbid media is also important for the preparation of dental restoration material. The visual impression under UV and Vis irradiation should match human teeth and depends on the emission, absorption and scattering properties of the material. The ideal energy dose in the photodynamic therapy strongly depends on the optical properties of human tissue, which become accessible with the presented experimental setup.

## Data Availability

The data that support the findings of this study are available from the corresponding author, J.J., upon reasonable request due to privacy.
